# Plasma Concentrations of BDNF and IGF-1 in Abstinent Cocaine Users with High Prevalence of Substance Use Disorders: Relationship to Psychiatric Comorbidity

**DOI:** 10.1371/journal.pone.0118610

**Published:** 2015-03-03

**Authors:** María Pedraz, Ana Isabel Martín-Velasco, Nuria García-Marchena, Pedro Araos, Antonia Serrano, Pablo Romero-Sanchiz, Juan Suárez, Estela Castilla-Ortega, Vicente Barrios, Rafael Campos-Cloute, Juan Jesús Ruiz, Marta Torrens, Julie Ann Chowen, Jesús Argente, Rafael de la Torre, Luis Javier Santín, María Ángeles Villanúa, Fernando Rodríguez de Fonseca, Francisco Javier Pavón

**Affiliations:** 1 Unidad Gestión Clínica de Salud Mental, Instituto de Investigación Biomédica de Málaga (IBIMA), Hospital Regional Universitario de Málaga-Universidad de Málaga, Málaga, Spain; 2 Department of Physiology, Faculty of Medicine, Complutense University of Madrid, Madrid, Spain; 3 Department of Pediatrics & Pediatric Endocrinology, Hospital Infantil Universitario Niño Jesús, Instituto de Investigación La Princesa, Madrid, Spain; 4 Department of Pediatrics, Universidad Autónoma de Madrid, Madrid, Spain; 5 Centro de Investigación Biomédica en Red de la Fisiopatología de la Obesidad y Nutrición (CIBEROBN), Instituto de Salud Carlos III, Madrid, Spain; 6 Centro de Tratamiento Ambulatorio Mijas Costa-Diputación de Málaga, Mijas, Spain; 7 Centro Provincial de Drogodependencia-Diputación de Málaga, Málaga, Spain; 8 Neurosciences Program, Institut Hospital del Mar d’Investigacions Mèdiques (IMIM), Barcelona, Spain; 9 Institut de Neuropsiquiatria i Addiccions (INAD) del Parc de Salut MAR, Barcelona, Spain; 10 Department of Psychiatry. Universitat Autònoma de Barcelona (UAB), Barcelona, Spain; 11 Facultat de Ciencies de la Salut i de la Vida, Universitat Pompeu Fabra (CEXS-UPF), Barcelona, Spain; 12 Departamento de Psicobiología y Metodología de las Ciencias del Comportamiento, Facultad de Psicología, Universidad de Málaga, Málaga, Spain; Case Western Reserve University School of Dental Medicine, UNITED STATES

## Abstract

Recent studies have identified biomarkers related to the severity and pathogenesis of cocaine addiction and common comorbid psychiatric disorders. Monitoring these plasma mediators may improve the stratification of cocaine users seeking treatment. Because the neurotrophic factors are involved in neural plasticity, neurogenesis and neuronal survival, we have determined plasma concentrations of brain-derived neurotrophic factor (BDNF), insulin-like growth factor 1 (IGF-1) and IGF-1 binding protein 3 (IGFBP-3) in a cross-sectional study with abstinent cocaine users who sought outpatient treatment for cocaine (*n* = 100) and age/body mass matched controls (*n* = 85). Participants were assessed with the diagnostic interview ‘Psychiatric Research Interview for Substance and Mental Disorders’. Plasma concentrations of these peptides were not different in cocaine users and controls. They were not associated with length of abstinence, duration of cocaine use or cocaine symptom severity. The pathological use of cocaine did not influence the association of IGF-1 with age observed in healthy subjects, but the correlation between IGF-1 and IGFBP-3 was not significantly detected. Correlation analyses were performed between these peptides and other molecules sensitive to addiction: BDNF concentrations were not associated with inflammatory mediators, lipid derivatives or IGF-1 in cocaine users, but correlated with chemokines (fractalkine/CX3CL1 and SDF-1/CXCL12) and N-acyl-ethanolamines (N-palmitoyl-, N-oleoyl-, N-arachidonoyl-, N-linoleoyl- and N-dihomo-γ-linolenoyl-ethanolamine) in controls; IGF-1 concentrations only showed association with IGFBP-3 concentrations in controls; and IGFBP-3 was only correlated with N-stearoyl-ethanolamine concentrations in cocaine users. Multiple substance use disorders and life-time comorbid psychopathologies were common in abstinent cocaine users. Interestingly, plasma BDNF concentrations were exclusively found to be decreased in users diagnosed with both primary and cocaine-induced disorders for mood and anxiety disorders. In summary, BDNF, IGF-1 and IGFBP-3 were not affected by a history of pathological use of cocaine supported by the absence of associations with other molecules sensitive to cocaine addiction. However, BDNF was affected by comorbid mood disorders. Further research is necessary to elucidate the role of BDNF and IGF-1 in the transition to cocaine addiction and associated psychiatric comorbidity.

## Introduction

Chronic cocaine use induces long-lasting neurochemical, structural and behavioral adaptive changes thought to result from altered gene and protein expression within cerebral areas playing a critical role in addiction and reward [1]. Some of these changes are not fully reversed upon prolonged abstinence, and may represent an example of aberrant cocaine-induced neuroplastic changes related to cocaine dependence and an increased susceptibility to relapse to drug taking even after a long period of abstinence [2,3].

Furthermore, long-term cocaine use is commonly associated with altered executive functions, impaired emotional processing capacity and a higher incidence of comorbid mental disorders, particularly mood and anxiety disorders [4,5,6]. The diagnosis of psychiatric comorbidity is an important consideration for effective therapies to overcome cocaine addiction. However, the accurate diagnosis of comorbid psychiatric disorders in cocaine addicts has to face two major problems, the effects of cocaine can conceal symptoms of other mental disorders and the diagnosis is defined by manifestations rather than by direct biomarkers [7,8].

Focusing on this last point, the search for peripheral biomarkers for both psychiatric and substance use disorders has caused an increasing interest in addiction psychiatry research over the last few years. This growing research has generated a number of putative biomarkers, mainly involving the immune system and inflammatory responses, which still require replication in larger studies [9,10,11]. Recently, our group has studied the plasma profile of inflammatory mediators and fatty acid derivatives in abstinent cocaine users under outpatient treatment [12,13]. We found certain cytokines and chemokines that might serve as reliable biomarkers for pathological use of cocaine (i.e., binge use and/or chronic use) and symptom severity [12]. Additionally, several fatty acid derivatives such as endocannabinoids and congeners were biomarkers for cocaine use disorders and psychiatric comorbidity [13].

Neurotrophic factors are peptides that are known for their role in mediating neuronal plasticity and neuronal growth. In addition to neurons, these factors are produced by other cells and can affect and integrate neural, immune and endocrine systems. They have been reported to mediate the effects of drugs for the treatment of mental disorders but also play a relevant role in the acute and chronic responses produced by addictive drugs [14]. We have focused this study on two trophic factors, which are found in plasma and involved in mediating neuronal plasticity, the brain-derived neurotrophic factor (BDNF) and the insulin-like growth factor 1 (IGF-1). In addition to IGF-1, we have also measured the IGF binding protein 3 (IGFBP-3) as relevant protein modulating its effects.

BDNF is a neurotrophin that participates in neuronal survival, differentiation, synaptogenesis and maintenance. Accumulating evidence suggests that alterations in the BDNF expression underlie a variety of psychiatric and neurological disorders. BDNF has been associated positively with some disorders (e.g., major depression and bipolar disorder) but there are also many non-specific or conflicting findings (schizophrenia and autism) [11,15]. Furthermore, BDNF has been also evaluated in cocaine addiction and comorbid disorders. Indeed, serum BDNF concentrations have been recently postulated as an indication of relapse risk during early recovery from cocaine dependence in a prospective study [16]. Another study performed by Corominas-Roso and colleagues showed that an increase in serum BDNF concentrations during early abstinence correlates with cocaine craving and abstinence symptoms [17], but interestingly these increased BDNF concentrations are not observed in patients displaying cocaine-induced psychosis [18].

IGF-1 is a trophic mediator regulated by growth hormone (GH) that may either be free or bound to binding proteins. IGFBP-3 is the most abundant binding protein in human blood and this complex prolongs the half-life of IGF-1. IGF-1 regulates proliferation, development and growth of neural cells (for recent reviews [19,20]). This peptide is also involved in the pathogenesis and evolution of psychiatric disorders in preclinical models [21,22] and clinical cases in old [23] and young [24] individuals. Thus, a cross-sectional study in older adults showed that the association between depressive symptoms and memory deficits is stronger with lower concentrations of circulating IGF-1 [23]. Young adults with GH deficiency exhibit anxious or depressed moods, which can be treated by GH therapy that increases IGF-1 concentrations. Furthermore, IGF-1 concentrations negatively correlate with depression, fatigue, tension and anxiety and positively with vigor and memory [24]. Regarding drugs of abuse in humans, IGF-1 concentrations have been assessed in alcohol and opiate dependence. Studies in alcohol dependents revealed a positive correlation between blood insulin level and alcohol craving, but not between alcohol craving and IGF-1 concentrations during either the active drinking phase or during abstinence [25]. In contrast to alcohol, serum IGF-1 is found to be elevated in opiate dependence [5].

The aim of the present cross-sectional study is to examine the plasma concentrations of BDNF, IGF-1 and IGFBP-3 in a cohort of abstinent cocaine users on an outpatient basis according to cocaine use history (duration of use, length of abstinence and cocaine symptom severity) and the comorbidity of other mental disorders. We found that plasma BDNF, IGF-1 and IGFBP-3 are unaltered in abstinent cocaine users but they are affected by the presence of comorbid mood and anxiety disorders.

## Methods and Materials

### 1 Subjects and recruitment

All participants in the present cross-sectional study were white Caucasians grouped into abstinent cocaine users and healthy controls. One-hundred and ten cocaine users were enrolled from outpatient treatment programs for cocaine addiction in the province of Málaga (Spain) for a 36 month- period (2011–2013). Eighty healthy individuals were recruited in parallel from a multidisciplinary staff working at the Hospital Regional Universitario de Málaga.

Cocaine users had to meet eligibility criteria based on inclusion and exclusion criteria. Inclusion criteria were as follows: ≥18 years to 65 years of age, intranasal cocaine use, diagnosis of a lifetime ‘pathological use’ of cocaine (chronic intoxication and/or binge), and abstinence from cocaine for at least 2 weeks before testing. The ‘pathological use’ of cocaine was determined through a psychiatric interview, while the abstinence of cocaine users was checked weekly by urine analysis in the outpatient treatment centers for cocaine addiction and plasma analyses [12]. Exclusion criteria were as follows: personal history of chronic diseases (e.g. cardiovascular, respiratory, renal, hepatic, neurological or endocrine diseases), personal history of cancer, infectious diseases, incapacitating cognitive alterations and/or severe schizophrenia, and pregnancy.

Controls were matched with the cocaine group for age and body mass index (BMI) and they were required to be ≥18 years to 65 years of age. In addition to the mentioned exclusion criteria for abstinent cocaine users, controls were excluded with: personal history of drug abuse and lifetime psychiatric disorders.

### 2 Clinical assessments

All cocaine users were evaluated according to ‘Diagnostic and Statistical Manual of Mental Disorders-4^th^ Edition-Text Revision’ (DSM-IV-TR) criteria, using the Spanish version of the ‘Psychiatric Research Interview for Substance and Mental Disorders’ (PRISM) [7,26]. Controls were initially evaluated by PRISM (for substance screening and abuse and dependence) and subsequently by the Spanish version of the ‘Composite International Diagnostic Interview’ (CIDI) for the detection of psychiatric disorders [27]. All the interviews were performed by experienced psychologists who had received both PRISM and CIDI training.

#### 2.1 Psychiatric Research Interview for Substance and Mental Diseases (PRISM)

The PRISM is a semi-structured interview that has demonstrated good psychometric properties in terms of test-retest reliability [28], inter-rater reliability [29] and validity [7] to diagnose psychiatric disorders among substance users.

Diagnoses were made using two time-frames: ‘current’ (criteria were met within the past year) and ‘past’ (criteria were met before the previous 12 months). Lifetime prevalence, taking into account both current and past diagnoses, was used to present the frequency of substance use disorders, non-substance use disorders and psychiatric comorbidity. In addition to the diagnoses of substance abuse and dependence, the PRISM differentiates ‘pathological use’ (chronic intoxication: substance use ≥4 days a week for ≥3 weeks; and/or binge use: ≥3 consecutive days of continuous substance use) from ‘occasional use’ (substance use less than 4 days a week, unless substance was used in a binge pattern).

The cocaine symptom severity was assessed combining the DSM-IV-TR criteria for cocaine use disorders: 7 dependence criteria (for diagnosis of dependence three or more co-occurring symptoms in a 12-month period are required); and 4 abuse criteria (one symptom is necessary for diagnosis of abuse), which is in agreement with the unidimensionality of DSM-5 criteria [30,31]. More details regarding analysis of cocaine symptom severity have been described previously (see [13]).

### 3 Laboratory methods for human samples

#### 3.1 Collection and analysis of plasma samples

Blood samples were obtained in the morning (09:00–11:00 h AM) after fasting for 8–12 h (previous to the psychiatric interviews). Venous blood was collected into 10 mL K_2_-EDTA tubes (BD, Franklin Lakes, NJ, USA) and processed to obtain plasma. Blood samples were centrifuged at 2,200×g for 15 min (4°C) and individually assayed for detecting infectious diseases by 3 rapid tests for HIV (Retroscreen HIV, QualPro Diagnostics-Tulip Group Ltd, Goa, India), hepatitis B (HBsAg Test, Toyo Diagnostics-Turklab Inc., Izmir, Turkey) and hepatitis C (Flaviscreen HCV, QualPro Diagnostics-Tulip Group Ltd, Goa, India). Samples testing positive were discarded following safety protocols.

Plasma analyses for cocaine metabolite (Benzoylecgonine Specific Direct ELISA Kit Immunalysis, Pomona, CA, USA) were performed to confirm cocaine abstinence. Four cocaine users who tested negative for drugs of abuse in urine analyses at the outpatient treatment centers for cocaine addiction were positive for benzoylecgonine in plasma, and these cocaine users were excluded from this study. Plasma samples were stored at -80°C until further analyses.

#### 3.2 Multiplex immunoassay analysis

A Bio-Plex Suspension Array System 200 (Bio-Rad Laboratories, Hercules, CA, USA) was used to quantify the plasma concentrations of anti- and pro-inflammatory cytokines, homeostatic and pro-inflammatory chemokines and BDNF following the manufacturer´s instructions as previously reported [12]. Human protein panels were used to simultaneously detect the following analytes: Tumor necrosis factor-alpha (TNFα); interleukin-1 beta (IL-1β); interleukin-6 (IL-6); interleukin-10 (IL-10); CX3CL1 [Chemokine (C-X_3_-C motif) ligand 1], commonly referred to as fractalkine; CCL2 [Chemokine (C-C motif) ligand 2], also referred to as monocyte chemotactic protein-1 (MCP-1); CXCL12 [Chemokine (C-X-C motif) ligand 12], also referred to stromal cell-derived factor-1 (SDF-1); and BDNF. Raw data (mean fluorescence intensity) were analyzed using the Bio-Plex Manager Software 4.1 (Bio-Rad Laboratories, Hercules, CA, USA). Data of plasma concentrations (pg of protein/mL) were used to perform multiple correlation studies and analyses of the means.

#### 3.3 Radioimmunoassay analysis for IGF1 and IGFBP-3

Plasma concentrations of total IGF-1 were estimated by double antibody radioimmunoassay (RIA), after removal of serum IGFBPs by acid-ethanol extraction. To confirm the removal of IGFBPs, extracted and non-extracted plasma fractions were incubated with ^125^I-IGF-1 at 4°C for 24 h. Dextran charcoal was used to separate the bound and free tracers. The IGF-1 antiserum (UB2–495) was a gift from Drs Underwood and Van Wisk distributed by the Hormone Distribution Program of the National Institute of Diabetes and Digestive and Kidney Diseases (NIDDK) through the National Hormone and Pituitary Program. Concentrations of IGF-1 were expressed in terms of rat IGF-1 from Gropep Bioreagents Pty Ltd (Adelaide, SA, Australia). Test sensitivity was 10 ng/mL and the intra-assay coefficient of variation was 8%. All samples were run in the same batch.

Plasma IGFBP-3 concentration was determined in duplicate by RIA using a commercially available kit (Mediagnost GmbH, Reutlingen, Germany) following the manufacturer´s protocol. The assay sensitivity was 500 pg/mL and the intra-assay coefficient of variation was 7.5%.

#### 3.4 Quantification of acyl derivatives

The following lipid derivatives and their respective deuterated forms were used for quantification: N-stearoyl-ethanolamine (SEA), N-palmitoyl-ethanolamine (PEA) and PEA-d_4_, N-oleoyl-ethanolamine (OEA) and OEA-d_4_, N-palmitoleoyl-ethanolamine (POEA), N-arachidonoyl-ethanolamine (AEA) and AEA-d_4_, N-linoleoyl-ethanolamine (LEA) and LEA-d_4_, N-docosahexaenoyl-ethanolamine (DHEA) and DHEA-d_4_, N-dihomo-γ-linolenoyl-ethanolamine (DGLEA), 2-arachidonoyl-glycerol (2-AG) and 2-AG-d_5_, and 2-linoleoyl-glycerol (2-LG). PEA-d_4_, OEA-d_4_ and AEA-d_4_ were used for quantification of POEA, SEA and DGLEA respectively, since their deuterated forms were not commercially available. All reagents were obtained from Cayman Chemical (Ann Arbor, MI, USA).

Sample extraction and the chromatographic separation were performed in a Liquid Chromatography-tandem Mass Spectrometry System (Agilent Technologies, Wilmington, DE, USA) as previously reported [13]. The tandem quadrupole mass spectrometer operated on the positive electrospray mode. The multiple reaction monitoring mode was used for the analysis with the following precursor to product ion transitions: m/z 328.1/62 for SEA, m/z 300.1/62 for PEA, m/z 304.4/66 for PEA-d_4_, m/z 326.1/62 for OEA, m/z 330.4/66 for OEA-d_4_, m/z 298.2/62 for POEA, m/z 348.3/62 for AEA, m/z 352.2/66 for AEA-d_4_, m/z 324.5/62 for LEA, m/z 328.5/66 for LEA-d_4_, m/z 372.6/62 for DHEA, m/z 376.3/66 for DHEA-d_4_, m/z 350.2/62 for DGLEA, m/z 379.2/287 for 2-AG, m/z 384.3/287 for 2-AG-d_5_ and m/z 355.2/263 for 2-LG. A six-point external calibration curve prepared in the mobile phase (10:90, A:B) and spiked with 0.4–25 ng of N-acyl-ethanolamines and 0.8–50 ng of 2-acyl-glycerols was used for the quantification [32]. Data of plasma concentration (ng of acyl derivative /mL) were used to perform multiple correlation studies.

### 4 Ethics statement

Written informed consent was obtained from each subject after they had received a complete description of the present study and had been given the chance to discuss any questions or issues. The study and protocols for recruitment were approved by the Ethics Committee of the Hospital Regional Universitario de Málaga (07/19/2009 PND049/2009 and PI0228–2013; CEI Provincial de Málaga) and therefore were conducted in accordance with the Declaration of Helsinki (seventh revision in 2013, Fortaleza, Brazil).

### 5 Statistical analyses

All data for graphs and tables are expressed as number and percentage of subjects [*n* (%)] or mean and standard deviation (SD) of concentrations [mean (SD)]. The significance of differences in categorical variables was determined by using the Fisher’s exact test; while continuous variables were evaluated by different statistical approaches according to the number of comparisons and the distribution of variables. For comparisons of two groups, the Student’s t-test was used for normally distributed continuous variables and the Mann-Whitney U test was used as non-parametric test. For comparisons of three or more groups, one-way and analysis of variance (ANOVA) with Bonferroni correction for multiple comparisons was used for normal distributions whereas the Kruskal—Wallis analysis with the Dunn’s post test was used as non-parametric analysis. Correlation analyses were performed by using the Pearson´s correlation coefficient (r) for continuous variables with normal distribution and Spearman´s rank correlation coefficient (rho) for continuous variables without normality and discrete variables. The Holm-Bonferroni correction was employed for multiple comparisons of correlation coefficients for controlling the type I errors. The normal distribution of variables was evaluated using the D’Agostino & Pearson omnibus normality test. Thresholds of 0.05 were applied for p-values and adjusted p-values. Statistical analyses were performed using the computer program Graph-Pad Prism version 5.04 (GraphPad Software, San Diego, CA, USA).

## Results

### 1 Sample demographics and clinical characteristics

A total of 185 subjects both sexes were selected for this study and grouped into the cocaine (*n* = 100) and control (*n* = 85) groups. The average participant was a 36–37 year-old male with a BMI of 26 (weighing 75–77 kg). A description of the sample is presented in Table 1.

**Table 1:** Baseline socio-demographic variables and lifetime psychiatric and substance use disorders.

VARIABLE		COCAINE GROUP	CONTROL GROUP	p-value
		*n* = 100	*n* = 85	
**AGE (≥18) [MEAN (SD)]**		35.8 (8.9)	37.3 (10.7)	0.299 ^**a**^
**SEX [*n* (%)]**	Women	18 (18.0)	25 (29.4)	0.081 ^**b**^
	Men	82 (82.0)	60 (70.6)	
**BODY MASS [MEAN (SD)]**	Body Mass Index	25.5 (4.5)	26.1 (4.0)	0.343 ^**a**^
	Weight (kg)	77.1 (14.4)	75.3 (11.0)	0.337 ^**a**^
**PSYCHOLOGICAL TREATMENT (EVER) [*n* (%)]**	No	67 (67.0)	80 (94.1)	**<0.001** ^**b**^
	Yes	35 (35.0)	5 (5.9)	
**SUBSTANCE USE TREATMENT (EVER) [*n* (%)]**	No	21 (21.0)	85 (100)	**<0.001** ^**b**^
	Yes	81 (81.0)	0 (0.0)	
**LIFETIME SUBSTANCE USE DISORDERS [*n* (%)]**	Cocaine	89 (89.0)	-	-
	Alcohol	64 (64.0)	-	
	Cannabis	23 (23.0)	-	
	Benzodiazepines	8 (8.0)	-	
	Heroin	8 (8.0)	-	
	Hallucinogens	6 (6.0)	-	
	Others	7 (7.0)	-	
**LIFETIME COCAINE USE DISORDERS [*n* (%)]**	Abuse or Dependence	89 (89.0)	-	-
	Abuse	78 (78.0)	-	
	Dependence	84 (84.0)	-	
**LIFETIME COMMON PSYCHIATRIC DISORDERS [*n* (%)]**	Mood Disorders	33 (33.0)	-	-
	Anxiety Disorders	22 (22.0)	-	
	Psychosis Disorders	13 (13.0)	-	
	Personality Disorders	31 (31.0)	-	

^a^ p-value from Student’s t-test.

^b^ p-value from Fisher’s exact test or Chi-square test.

Cocaine users displayed cocaine abstinence for 234.7 (436.3) days [mode: 30 days (range: 2,555)]. The percentages of cocaine users treated for substance use and psychological condition were 81.0% and 35.0% respectively. In contrast, only 17.6% of controls received psychological treatments.

Cocaine use disorders were the most prevalent lifetime substance use disorders (89%) followed by lifetime alcohol (64%), cannabis (23%), benzodiazepines (8%) and heroin (8%) use disorders (not including caffeine or nicotine).

Regarding common psychiatric disorders assessed with the PRISM, we found high prevalences of comorbid mood (33%), anxiety (22%), psychosis (13%) and personality (31%) disorders. Therefore, a vast majority of cocaine users who participated in this study were diagnosed with substance use disorders or multiple substance use disorders and psychiatric comorbidity.

### 2 Plasma concentrations of BDNF, IGF-1 and IGFBP-3

Mean plasma concentrations for BDNF, IGF-1 and IGFBP-3 were similar in both groups. The plasma concentrations of each protein were as follows: 274.9±200.3 pg/mL and 269.4±242.7 pg/mL for BDNF, 212.2±63.9 ng/mL and 210.7±51.2 ng/mL for IGF-1, and 3.91±0.94 μg/mL and 3.78±0.93 μg/mL for IGFBP-3, in the cocaine and control groups respectively.

#### 2.1 Plasma concentrations of BDNF, IGF-1 and IGFBP-3 in relation to sex

**Fig 1:**
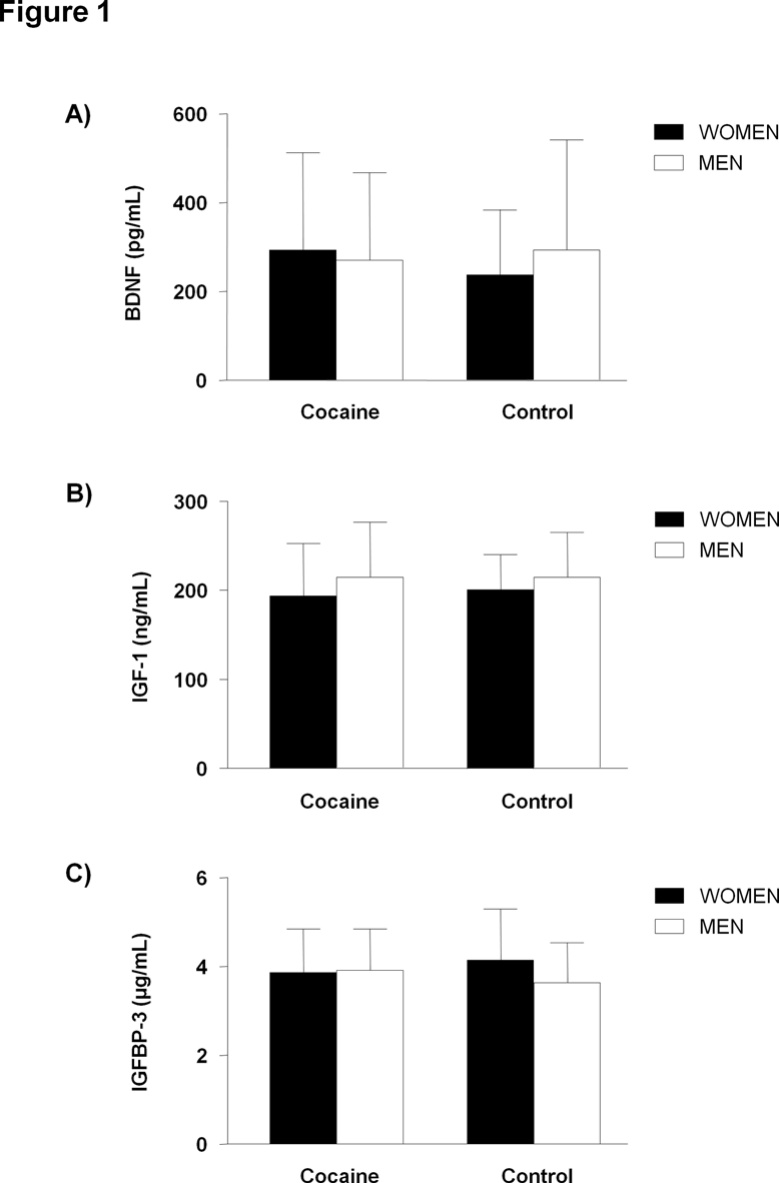
Plasma concentrations of BDNF, IGF-1 and IGFBP-3 according to sex in abstinent cocaine users and control subjects. **A)** BDNF (pg/mL); **B)** IGF-1 (ng/mL); and **C)** IGFBP-3 (μg/mL). Bars are the means and SD. Data were analyzed by two-way analyses (cocaine use [cocaine group and control group] and sex [women and men]).

As indicated in Fig. 1, we examined whether the sex composition in the cocaine and control groups affects the plasma concentrations of BDNF, IGF-1 and IGFBP-3. A two-way ANOVA was performed taking into consideration cocaine use and sex as factors. We did not observe main effects or interaction of these factors on the concentrations of BDNF, IGF-1 and IGFBP-3 between men and women in both groups.

#### 2.2 Plasma concentrations of BDNF, IGF-1 and IGFBP-3 in relation to age

**Fig 2:**
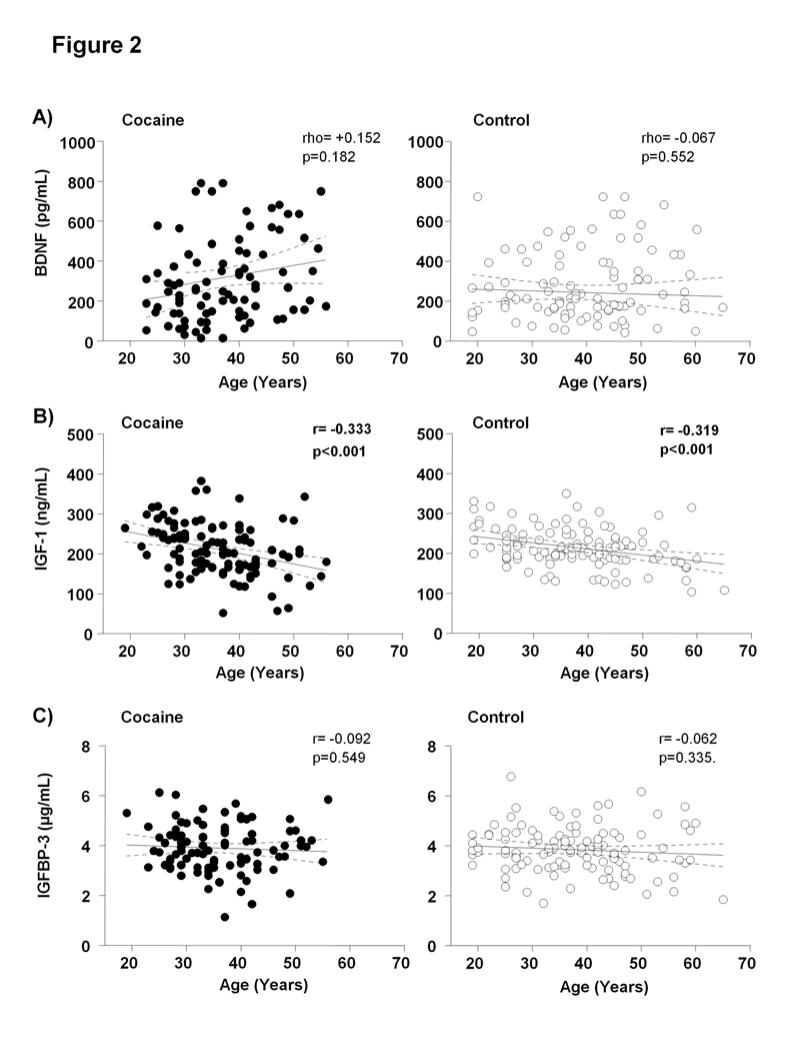
Correlation analyses between plasma concentrations of BDNF, IGF-1 and IGFBP-3 and age in abstinent cocaine users (black circles) and control subjects (white circles). **A)** BDNF (pg/mL); **B)** IGF-1 (ng/mL); and **C)** IGFBP-3 (μg/mL). Dots are individual values. (r) Pearson´s correlation coefficient; (rho) Spearman´s correlation coefficient; (p) p-value for statistical significance.

The influence of age on growth factors has been extensively described, especially with IGF-1 [33,34,35]. Correlation analyses were performed between plasma concentrations of BDNF, IGF-1 or IGFBP-3 and age for each study group, as shown in Fig. 2. The plasma IGF-1 concentrations were negatively correlated with age in the cocaine (r = -0.33, p<0.001) and control (r = -0.32, p<0.001) groups using the Pearson’s correlation coefficient for normal distributions. These correlations were not significant for BDNF and IGFBP-3 concentrations.

### 3 Multiple correlation analyses of BDNF, IGF-1 and IGFBP-3 with other plasma molecules sensitive to cocaine addiction

We determined the degree of association among these peptides (BDNF, IGF-1 and IGFBP-3) and other plasma mediators in the cocaine and control groups. Recently, we have reported that certain circulating pro-inflammatory mediators and fatty acid derivatives are influenced by cocaine addiction [12,13]. Thus, we examined the degree of association of BDNF, IGF-1 and IGFBP-3 with these molecules and among themselves in the sample. The significances of the resulting correlation coefficients were statistically corrected using the Holm-Bonferroni method to counteract the problem of multiple comparisons for the control and cocaine groups.

#### 3.1 BDNF

Plasma concentrations of BDNF showed a non-normal distribution in abstinent cocaine subjects and controls and the correlation coefficients calculated were rho (Table 2).

**Table 2:** Multiple correlations between plasma concentrations of BDNF and other plasma molecules in the cocaine group.

**MULTIPLE CORRELATION ANALYSIS** ^**1**^ ^,^ ^**2**^						
**VARIABLE**	**BDNF**					
	**COCAINE**			**CONTROL**		
	**rho**	**p-value**	**adjusted p-value**	**rho**	**p-value**	**adjusted p-value**
**CX**_**3**_ **CL1 (fractalkine)**	-0.094	0.441	ns	**+0.693**	**<0.001**	**<0.001**
**CCL2 (MCP-1)**	-0.005	0.968	ns	+0.009	0.941	ns
**CXCL12 (SDF-1)**	-0.110	0.363	ns	**+0.784**	**<0.001**	**<0.001**
**IL1β**	-0.109	0.372	ns	-0.135	0.269	ns
**TNFα**	-0.133	0.272	ns	-0.189	0.120	ns
**IL6**	-0.260	0.029	ns	+0.054	0.660	ns
**IL10**	-0.110	0.365	ns	+0.031	0.799	ns
**SEA**	-0.047	0.695	ns	+0.325	**0.007**	ns
**PEA**	-0.058	0.629	ns	**+0.492**	**<0.001**	**0.003**
**OEA**	-0.133	0.270	ns	**+0.400**	**<0.001**	**0.033**
**POEA**	-0.037	0.760	ns	+0.331	**0.006**	ns
**AEA**	-0.150	0.213	ns	**+0.447**	**<0.001**	**0.005**
**LEA**	-0.187	0.119	ns	**+0.423**	**<0.001**	**0.014**
**DGLEA**	-0.170	0.156	ns	**+0.524**	**<0.001**	**0.002**
**DHEA**	+0.009	0.938	ns	+0.301	**0.012**	ns
**2-AG**	+0.074	0.538	ns	+0.054	0.661	ns
**2-LG**	-0.056	0.640	ns	+0.122	0.320	ns
**IGF-1**	-0.157	0.210	ns	-0.111	0.291	ns
**IGFBP-3**	-0.170	0.156	ns	-0.221	0.106	ns

^**1**^ All variables were assessed for normality to select the adequate correlation coefficient (r; rho).

^**2**^ Adjusted p-values were calculated using Holm-Bonferroni correction (3x18 correlations per group).

Abbreviations: ns, non-significant.

In the cocaine group, BDNF concentrations did not correlate with the concentrations of chemokines (CX_3_CL1, CCL2 and CXCL12), cytokines (IL1β, TNFα, IL6 and IL10), fatty acid derivatives (N-acyl-ethanolamines and 2-acyl-glycerols), IGF-1 or IGFBP-3. In contrast, BDNF concentrations correlated positively with the concentrations of certain chemokines and N-acyl-ethanolamines in the control group. Concretely, BDNF significantly correlated with CX_3_CL1 (rho = +0.69, adjusted p<0.001) and CXCL12 (rho = +0.78, adjusted p<0.001); PEA (rho = +0.49, adjusted p<0.01), OEA (rho = +0.40, adjusted p<0.05), AEA (rho = +0.45, adjusted p<0.01), LEA (rho = +0.42, adjusted p<0.05) and DGLEA (rho = +0.52, adjusted p<0.01). Although BDNF was initially associated with other N-acyl-ethanolamines (SEA, POEA and DHEA), the statistical adjustment for multiple comparisons rejected them.

#### 3.2 IGF-1

Plasma concentrations of IGF-1 were normally distributed in the cocaine and control groups and Pearson´s correlation coefficients (r) were calculated for all comparisons. As shown in Table 3, IGF-1 concentrations did not correlate with the concentrations of the plasma molecules that were assessed in the cocaine group. Only we found an initial association with its binding protein IGFBP-3 prior to apply the correction test that was rejected. However, in the control group this positive correlation between the concentrations of IGF-1 and IGFBP-3 was significant after adjusting the significance (r = +0.46, adjusted p<0.01). Similar to BDNF, IGF-1 showed correlations with some N-acyl-ethanolamines (PEA and OEA) that were also discarded after correcting them.

**Table 3:** Multiple correlations between plasma concentrations of IGF-1 and other plasma molecules in the cocaine group.

**MULTIPLE CORRELATION ANALYSIS** ^**1**^ ^**,**^ ^**2**^						
**VARIABLE**	**IGF-1**					
	**COCAINE**			**CONTROL**		
	**r**	**p-value**	**adjusted p-value**	**r**	**p-value**	**adjusted p-value**
**CX**_**3**_ **CL1 (fractalkine)**	-0.062	0.608	ns	-0.062	0.792	ns
**CCL2 (MCP-1)**	+0.087	0.472	ns	+0.087	0.137	ns
**CXCL12 (SDF-1)**	+0.013	0.914	ns	+0.013	0.845	ns
**IL1β**	+0.067	0.582	ns	+0.067	0.180	ns
**TNFα**	+0.048	0.693	ns	+0.048	0.638	ns
**IL6**	+0.078	0.519	ns	+0.078	0.520	ns
**IL10**	+0.089	0.462	ns	+0.089	0.799	ns
**SEA**	+0.086	0.436	ns	+0.086	0.187	ns
**PEA**	+0.054	0.624	ns	-0.281	**0.019**	ns
**OEA**	-0.064	0.560	ns	-0.264	**0.028**	ns
**POEA**	-0.142	0.195	ns	+0.006	0.964	ns
**AEA**	-0.085	0.437	ns	-0.186	0.125	ns
**LEA**	-0.095	0.387	ns	-0.133	0.275	ns
**DGLEA**	-0.018	0.873	ns	-0.184	0.131	ns
**DHEA**	+0.171	0.118	ns	-0.309	0.010	ns
**2-AG**	-0.120	0.275	ns	-0.097	0.430	ns
**2-LG**	-0.074	0.503	ns	-0.119	0.331	ns
**IGFBP-3**	+0.327	**0.006**	ns	**+0.463**	**<0.001**	**0.003**

^**1**^ All variables were assessed for normality to select the adequate correlation coefficient (r; rho).

^**2**^ Adjusted p-values were calculated using Holm-Bonferroni correction (3x18 correlations per group).

Abbreviations: ns, non-significant.

#### 3.3 IGFBP-3

As indicated in Table 4, IGFBP-3 concentrations passed the normality test in both groups and Pearson´s correlation coefficients (r) were calculated. In the cocaine group, plasma concentrations of IGFBP-3 were not associated with the plasma mediators that were examined with the exception of SEA. Thus, plasma concentrations of IGFBP-3 correlated positively with SEA (r = +0.40; adjusted p<0.05). Regarding control subjects, IGFBP-3 concentrations were not correlated with the rest of molecules although some positive associations were observed in paired-comparisons with cytokines (IL1β, IL6 and IL10) without adjustment for multiple comparisons.

**Table 4:** Multiple correlations between plasma concentrations of IGFBP-3 and other plasma molecules in the cocaine group.

**MULTIPLE CORRELATION ANALYSIS** ^**1**^ ^**,**^ ^**2**^						
**VARIABLE**	**IGFBP-3**					
	**COCAINE**			**CONTROL**		
	**r**	**p-value**	**adjusted p-value**	**r**	**p-value**	**adjusted p-value**
**CX**_**3**_ **CL1 (fractalkine)**	-0.006	0.963	ns	+0.113	0.356	ns
**CCL2 (MCP-1)**	+0.126	0.303	ns	+0.171	0.159	ns
**CXCL12 (SDF-1)**	-0.113	0.357	ns	+0.141	0.250	ns
**IL1β**	-0.062	0.611	ns	+0.317	**0.008**	ns
**TNFα**	-0.047	0.699	ns	+0.233	0.054	ns
**IL6**	+0.067	0.587	ns	+0.321	**0.007**	ns
**IL10**	-0.052	0.672	ns	+0.244	**0.044**	ns
**SEA**	**+0.397**	**<0.001**	**0.011**	-0.091	0.459	ns
**PEA**	+0.216	0.058	ns	-0.048	0.696	ns
**OEA**	-0.226	0.056	ns	+0.115	0.348	ns
**POEA**	-0.121	0.272	ns	+0.046	0.710	ns
**AEA**	-0.079	0.473	ns	+0.125	0.307	ns
**LEA**	+0.038	0.732	ns	-0.003	0.979	ns
**DGLEA**	-0.084	0.446	ns	+0.006	0.963	ns
**DHEA**	+0.042	0.707	ns	+0.079	0.521	ns
**2-AG**	+0.010	0.926	ns	+0.145	0.235	ns
**2-LG**	+0.093	0.398	ns	+0.132	0.280	ns

^**1**^ All variables were assessed for normality to select the adequate correlation coefficient (r; rho).

^**2**^ Adjusted p-values were calculated using Holm-Bonferroni correction (3x18 correlations per group).

Abbreviations: ns, non-significant.

Overall, while BDNF concentrations correlated positively with chemokines and N-acyl-ethanolamines in the control group, IGFBP-3 was found to be positively associated only with SEA in the cocaine group. Moreover, IGF-1 and IGFBP-3 correlated positively in the control group but not in the cocaine group.

### 4 Plasma concentrations of BDNF, IGF-1 and IGFBP-3 in relation to variables associated with cocaine use

In the cocaine group, correlation analyses were performed between plasma concentrations of BDNF, IGF-1 or IGFBP-3 and length of cocaine abstinence (days) and duration of cocaine use (years) (S1 Fig.). Because both length of cocaine abstinence and duration of cocaine were variables with a non-normal distribution, Spearman’s rank correlation coefficients (rho) were used for determining associations. However, we observed no correlations between these mediators and variables related to abstinence and cocaine use.

Additional correlation analyses were performed between plasma concentrations of BDNF, IGF-1 or IGFBP-3 and individual scores of DSM-IV-TR criteria for cocaine abuse and dependence. The concentrations of these peptides were not associated with the cocaine symptom severity (S2 Fig.). As expected, the cocaine group displayed an increased number of criteria for cocaine use disorders (7.3±3.3 criteria) which indicates a high cocaine symptom severity.

### 5 Psychiatric comorbidity and substance use disorders

Because all outpatient cocaine users display high rates of comorbid psychiatric disorders, we evaluated plasma concentrations of BDNF, IGF-1 and IGFBP-3 in the most common psychiatric comorbidities among substance users, as shown in Table 5.

**Table 5:** Plasma concentrations of BDNF, IGF-1 and IGFBP-3 in abstinent cocaine users grouped by diagnosis of common psychiatric disorders in substance users.

**VARIABLE**				
**PSYCHIATRIC DISORDER**	***n* (%)**	**BDNF pg/mL [mean (SD)]**	**IGF-1 ng/mL [mean (SD)]**	**IGFBP-3 μg/mL [mean (SD)]**
**MOOD DISORDERS** ^**1**^	33 (33.0)	240.2 (177.4)	213.1 (83.4)	3.83 (0.99)
Primary	11 (11.0)	297.0 (223.2)	246.4 (59.7)	4.31 (0.73)
Cocaine-induced	17 (18.0)	227.6 (150.4)	196.5 (91.8)	3.83 (1.07)
Primary & Cocaine-induced	5 (5.0)	**131.8 (66.7)** ^**a**^	186.5 (38.8)	3.30 (0.67)
**NO MOOD DISORDERS**	67 (67.0)	294.9 (211.4)	210.8 (52.5)	3.87 (0.92)
**ANXIETY DISORDERS**	22 (22.0)	268.2 (171.1)	191.6 (61.9)	3.80 (0.91)
Primary	12 (12.0)	278.9 (170.0)	182.3 (67.6)	3.84 (0.87)
Cocaine-induced	7 (7.0)	293.6 (199.3)	199.9 (67.2)	4.11 (1.09)
Primary & Cocaine-induced	3 (3.0)	**137.9 (0.9)** ^**b**^ ^**,**^ *	209.8 (16.0)	3.16 (0.75)
**NO ANXIETY DISORDERS**	78 (78.0)	276.8 (208.9)	217.1 (63.7)	3.94 (0.95)
**PSYCHOTIC DISORDERS**	13 (13.0)	262.9 (209.9)	206.7 (59.3)	3.83 (0.84)
Primary	2 (2.0)	163.4 (168.1)	178.5 (0.0)	3.49 (0.30)
Cocaine-induced	11 (11.0)	281.0 (218.4)	211.0 (62.9)	3.89 (0.90)
Primary & Cocaine-induced	-	-	-	-
**NO PSYCHOTIC DISORDERS**	87 (87.0)	277.2 (199.9)	213.0 (64.8)	3.92 (0.95)
**PERSONALITY DISORDERS (CLUSTER-B)** ^**2**^	31 (31.0)	232.8 (176.8)	197.0 (64.5)	3.83 (0.91)
**NO PERSONALITY DISORDERS**	69 (69.0)	294.5 (208.9)	218.1 (63.4)	3.94 (0.95)
**CONTROL GROUP**		**269.4 (242.7)**	**210.7 (51.2)**	**3.78 (0.93)**

^**1**^ Mood disorders include major depressive disorder, dysthymic disorder, bipolar disorders (mania and hypomania) and cocaine-induced mood disorders.

^**2**^ Cluster B personality disorders include borderline and antisocial personality disorders.

^**a**^ p<0.05 denotes significant differences compared to the *no mood disorders* subgroup.

^**b**^ p<0.05 denotes significant differences compared to the *no anxiety disorders* subgroup.

* p<0.05 denotes significant differences compared to the control group.

The DSM-IV-TR Axis I disorders included mood disorders, anxiety and psychosis. Considering the exclusive diagnoses, the prevalences of cocaine-induced mood and psychotic (18.0% and 11.0% respectively) disorders were higher than the primary mood and psychotic (11.0% and 2.0% respectively) disorders, unlike cocaine-induced and primary anxiety disorders (7.0% and 12.0% respectively). Also, some cocaine users were diagnosed with both primary and cocaine-induced mood (*n* = 5) and anxiety (*n* = 3) disorders. With respect to personality disorders, the prevalence reached a 31.0%.

Considering the plasma concentrations of BDNF, IGF.1 and IFGBP-3 in these outpatient users diagnosed with psychiatric comorbidities, we only observed significant changes in BDNF concentrations. Concretely, abstinent cocaine users diagnosed with both primary and cocaine-induced disorders (for mood or anxiety disorders) displayed significant decreases in plasma BDNF concentrations (p<0.05) compared to those users with no mood disorders or no anxiety. Additionally, the decrease in BDNF concentrations in subjects diagnosed with both primary and cocaine-induced anxiety disorders were also significant (p<0.05) relative to the control group.

Regarding IGF-1 and IGFBP-3, we observed no changes in their plasma concentrations by the presence of psychiatric comorbidities.

## Discussion

The present exploratory and cross-sectional study found that the plasma concentrations of BDNF and IGF-1 are unaltered by a lifetime pathological use of cocaine in abstinent cocaine users under treatment. Indeed, plasma concentrations of these factors were not influenced by the length of abstinence, duration of cocaine use or cocaine symptom severity. The association of plasma IGF-1 concentrations with age was not affected by the pathological use of cocaine, although the association of IGF-1 and IGFBP-3 did not reach statistical significance after correcting it in abstinent cocaine users. Additionally, the correlations of BDNF concentrations with chemokines and N-acyl-ethanolamines in the control group were not observed in cocaine subjects. However, we detected a positive correlation between the concentrations of IGFBP-3 and SEA in the cocaine group. On the other hand, we found an elevated prevalence of comorbid psychiatric disorders in these patients with polysubstance use and changes in BDNF concentrations related to the diagnosis of mood and anxiety disorders. Thus, plasma BDNF concentrations were reduced in cocaine users diagnosed with both primary and cocaine-induced mood and anxiety disorders.

### BDNF in abstinent cocaine users

Although we found no changes in BDNF concentrations, several clinical studies have reported changes in the serum BDNF concentrations of cocaine dependents during abstinence, suggesting that BDNF is a reliable biomarker for cocaine addiction. A first study in cocaine dependent individuals found that BDNF concentrations are increased during early abstinence, and the elevated BDNF is predictive of relapse risk during early recovery from cocaine dependence [16]. Related to this finding, another study reported that BDNF concentrations are positively correlated with cocaine craving and abstinence symptoms [17]. Similarly, more recent studies in crack cocaine dependent individuals have found high blood BDNF concentrations during early abstinence [36] and negative correlations with severity [37] and amount of cocaine used [38]. All these prospective studies were conducted in cocaine addicts following inpatient detoxification treatments during 2–4 weeks, unlike our study that is a cross-sectional study in cocaine users seeking treatment in outpatient programs. Consequently, we have a single measure for BDNF from each cocaine user, variable periods of abstinence and uncontrolled environmental factors (inherent in outpatient treatments) that may be interfering with the present data.

### IGF-1 and IGFBP-3 in abstinent cocaine users

Numerous studies in mammals have reported that IGF-1 concentrations decline with advancing age, showing the association of IGF-1 with longevity and age-related diseases (e.g., cancer, cardiovascular disease, diabetes, osteoporosis, and neurodegenerative diseases) [39,40,41]. We observed that IGF-1 concentrations correlated significantly with the age of the participants. According to the present results, the history of a pathological use of cocaine did not influence the association of IGF-1 concentrations and aging because the significant and negative correlation between both variables was identical to the control group. However, we did not evaluated the cognitive impairment in the sample [42].

Focusing on cocaine addiction, we observed no changes in the plasma concentrations of IGF-1 and IGFBP-3 of abstinent users and concentrations did not correlated with addiction-related variables such as severity cocaine symptom, abstinence or duration of cocaine use. We did not find literature about an association between IGF-1 and cocaine but other drugs of abuse have been investigated. Studies in rodents suggest that IGF-1 is altered in cerebral areas related to the development of addiction after chronic exposure to morphine [43,44]. In humans, a recent study in patients with opiate use dependence has demonstrated that serum IGF-1 is elevated [5]. In addition to opiates, various studies evaluating IGF-1 have been conducted in alcohol dependent subjects but the authors did not observe any interaction between alcohol addiction and this peptide in blood or brain [25,45]. However, a recent study in alcohol dependent patients has found that IGF-1 might play a role in the cognitive function in these subjects [46].

### Lack of association with biomarkers of cocaine addiction

Overall, the lack of influence of cocaine addiction on plasma concentrations of BDNF and IGF-1 was confirmed through multiple analyses of correlation coefficients of these factors with other circulating molecules sensitive to cocaine addiction and/or psychiatric comorbidity in cocaine abstinent subjects from similar observational studies [12,13]. Chemokines and pro-inflammatory mediators are affected by the cocaine symptom severity, while anti-inflammatory fatty acid derivatives such as endocannabinoids and their congeners are affected by the history of pathological use of cocaine and the presence of comorbid disorders. Thus, the significant correlations between these cocaine-sensitive molecules and BDNF and IGF-1 (and IGFBP-3) in controls vanished in the cocaine group. However, we observed only one significant correlation after multiple comparisons in the cocaine group, in particular IGFBP-3 and SEA concentrations, but the SEA concentrations is not affected by cocaine use unlike other N-acyl-ethanolamines [13].

Because IGF-1 forms a ternary complex with IGFBP-3 [47], we also studied the association between both peptides. We detected a positive correlation between plasma concentrations of IGF-1 and IGFBP-3 in the control participants, but such association was weakly affected by the lifetime pathological use of cocaine.

### BDNF and IGF-1 in abstinent cocaine users with psychiatric comorbidity

Similar to these previous cross-sectional studies in abstinent cocaine users recruited from outpatient programs, we have detected a high prevalence of comorbid psychiatric disorders, approximately 60% [12,13]. Although these plasma peptide concentrations were unaltered in cocaine users, we examined these concentrations according to the diagnosis of primary and cocaine-induced disorders. Substantial evidence indicates neurotrophic/growth factors such as BDNF and IGF-1 are involved in the pathogenesis of common psychiatric disorders. We have indeed observed in this study changes in circulating concentrations of these neurotrophic factors in mood and anxiety disorders, especially in BDNF.

### BDNF in psychiatric comorbidity

Current literature on BDNF and mental disorders is particularly focused on depressive and bipolar disorders. Thus, circulating concentrations of BDNF are decreased in depressed patients compared to controls and that they increase significantly with antidepressant treatment [48,49,50]. The reduction of plasma BDNF concentration has also been related to suicidal behavior in major depression [51] whereas other studies in bipolar patients have found a significant association with the severity of depression [52,53], suggesting that plasma BDNF concentrations may be a marker of depression and/or bipolar disorder. Our findings are consistent with the literature because BDNF concentrations in cocaine users diagnosed with mood disorders, both primary and cocaine-induced, were found to be decreased. However, only five cocaine users were identified with this complicated diagnosis and, therefore, the statistical significance of this reduction may be called into question. We observed no differences in the BDNF concentrations of those abstinent subjects displaying primary mood disorders or cocaine-induced mood disorders separately.

BDNF has also emerged as a potential biomarker for anxiety from preclinical studies in rodents [54]. Translational studies have reported that early stress correlates negatively with peripheral BDNF concentrations later in life [55]. A recent review of clinical studies showed that BDNF concentrations were lower in individuals with any anxiety disorder compared to those without anxiety but this is not consistent across the literature [56]. In support of this, abstinent cocaine users diagnosed with both primary and cocaine-induced anxiety disorders exhibited a significant decrease in plasma BDNF, as seen previously with mood disorders. Again, the main limitation of this observation is related to the reduced number of individuals with this dual (primary and cocaine-induced anxiety) comorbid diagnosis.

### IGF-1 in psychiatric comorbidity

Several studies in old and young populations have shown the association between IGF-1 concentrations and depressive symptoms [23,24]. It has been reported that plasma IGF-1 concentrations are increased in acute depressed patients [57] and similarly, another study demonstrated that patients with bipolar disorder have elevated IGF-1 concentrations [58]. Although our data are consistent with these observations and cocaine users with primary mood disorders displayed an increase in plasma IGF-1 and IGFBP-3 concentrations, these increases did not reach statistical significance relative to the control group and abstinent users without mood disorders.

We need to perform additional studies to establish the mechanisms of action of these trophic factors (especially BDNF) and the selectivity in the influence of each factor by a specific comorbid disorder in cocaine addiction.

### Limitations and future perspectives

Although our findings support the importance of monitoring BDNF and IGF-1 in the context of cocaine addiction with psychiatric comorbidity, we are aware of the limitations of the present exploratory study. Firstly, the number of cases reported in our study with comorbid disorders is small and the replication is necessary. We cannot conclude whether these changes in BDNF and IGF-1 concentrations are exclusive to cocaine addiction or not because new studies in psychiatric patients with no history of drug use will be necessary to elucidate their role in mental disorders. Additional studies to determine plasma BDNF and IGF-1 in active cocaine users are necessary to confirm the lack of effects produced by the presence of cocaine on circulating concentrations of these factors. Further, longitudinal studies during cocaine abstinence could indicate whether these concentrations are unaltered or time-dependent.

The present study was conducted on outpatient subjects and, therefore we tried to show a realistic example of individuals seeking treatment in public centers for addiction but without being isolated from their social and familiar context. Nevertheless, from our results, we believe that monitoring neurotrophic factors such as BDNF and IGF-1 in cocaine users seeking treatment remains to be investigated to further improve the stratification of these patients taking into consideration the psychiatric comorbidities.

## Supporting Information

S1 Fig Correlation analyses between plasma concentrations of BDNF, IGF-1 and IGFBP-3 and variables related to addiction: length of abstinence and amount of cocaine use in abstinent cocaine users. **A)** BDNF (pg/mL); **B)** IGF-1 (ng/mL); and **C)** IGFBP-3 (μg/mL). Black dots are individual values. (r) Pearson´s correlation coefficient; (rho) Spearman´s correlation coefficient; (p) p-value for statistical significance.
(TIF)

S2 Fig Correlation analyses between plasma concentrations of BDNF, IGF-1 and IGFBP-3 and total number of DSM-IV-TR criteria for cocaine abuse and dependence in abstinent cocaine users. **A)** BDNF (pg/mL); **B)** IGF-1 (ng/mL); and **C)** IGFBP-3 (μg/mL). Black dots are individual values. (rho) Spearman´s correlation coefficient; (p) p-value for statistical significance.
(TIF)
