# Supplementary figures and images for: Plasma Concentrations of BDNF and IGF-1 in Abstinent Cocaine Users with High Prevalence of Substance Use Disorders: Relationship to Psychiatric Comorbidity
Source: PLoS One. 2015 Mar 3;10(3):e0118610. doi: 10.1371/journal.pone.0118610 (PMC4348520; doi:10.1371/journal.pone.0118610)

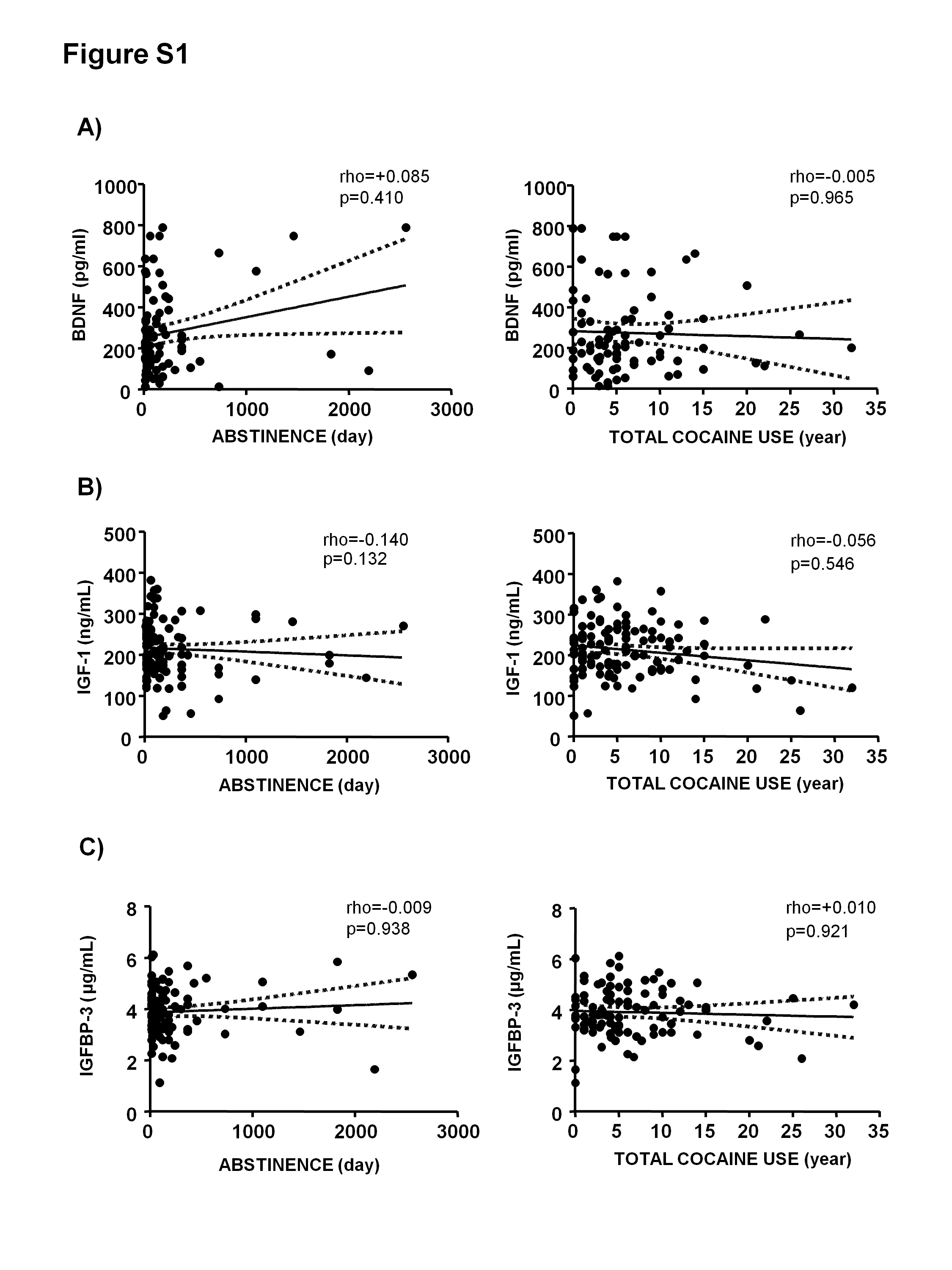

Supplement: S1 Fig — A) BDNF (pg/mL); B) IGF-1 (ng/mL); and C) IGFBP-3 (μg/mL). Black dots are individual values. (r) Pearson´s correlation coefficient; (rho) Spearman´s correlation coefficient; (p) p-value for statistical significance. (TIF) [file pone.0118610.s001.tif]

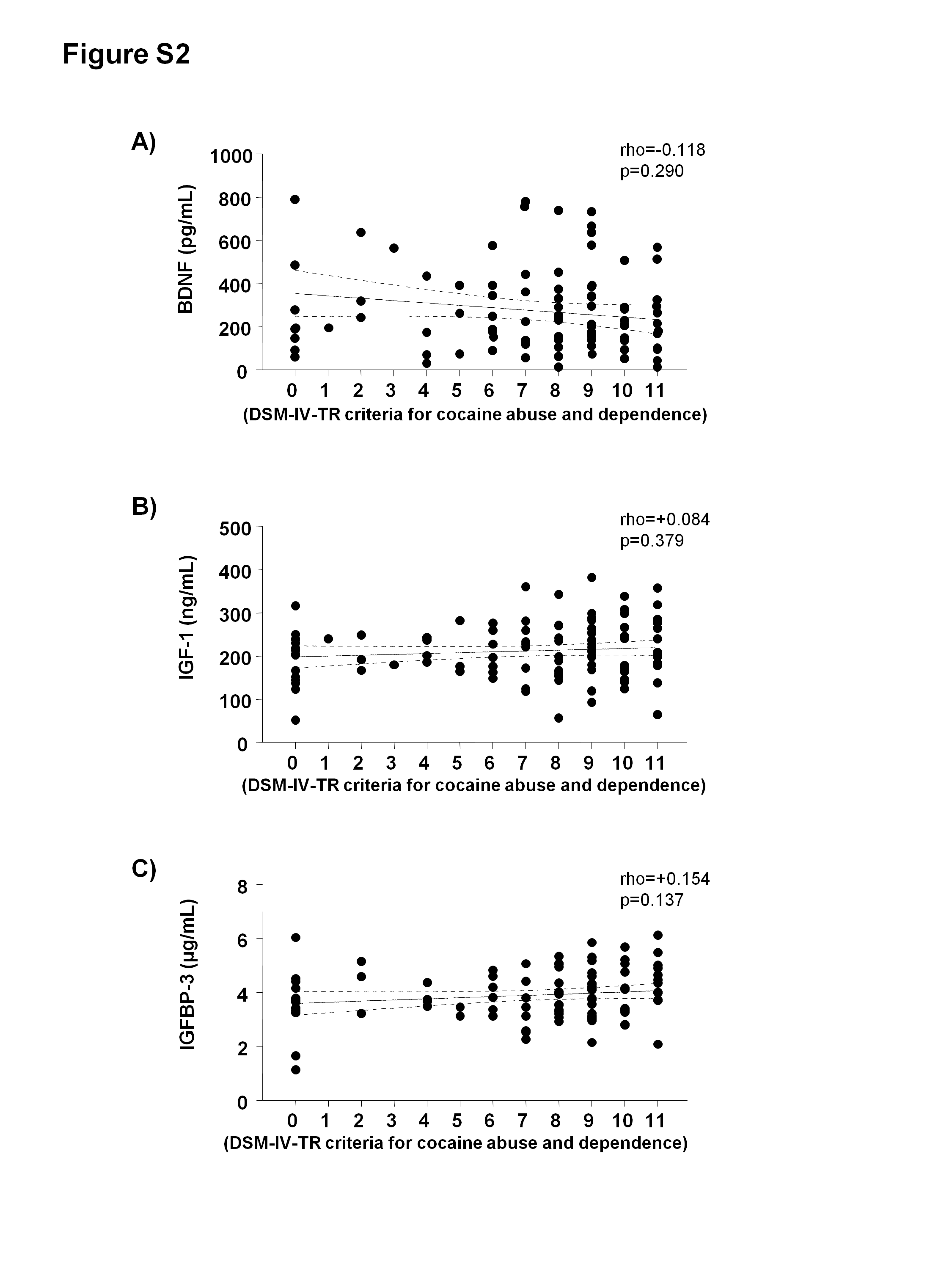

Supplement: S2 Fig — A) BDNF (pg/mL); B) IGF-1 (ng/mL); and C) IGFBP-3 (μg/mL). Black dots are individual values. (rho) Spearman´s correlation coefficient; (p) p-value for statistical significance. (TIF) [file pone.0118610.s002.tif]
